# Recent advances of γ-aminobutyric acid: Physiological and immunity function, enrichment, and metabolic pathway

**DOI:** 10.3389/fnut.2022.1076223

**Published:** 2022-12-22

**Authors:** Zhou Heli, Chen Hongyu, Bao Dapeng, Tan Yee Shin, Zhong Yejun, Zhang Xi, Wu Yingying

**Affiliations:** ^1^School of Health Science and Engineering, University of Shanghai for Science and Technology, Shanghai, China; ^2^National Engineering Research Center of Edible Fungi, Key Laboratory of Applied Mycological Resources and Utilization of Ministry of Agriculture, Institute of Edible Fungi, Shanghai Academy of Agricultural Sciences, Shanghai, China; ^3^Faculty of Science and Mushroom Research Centre, Institute of Biological Sciences, University of Malaya, Kuala Lumpur, Malaysia; ^4^State Key Laboratory of Food Science and Technology, Nanchang University, Nanchang, Jiangxi, China; ^5^BannerBio Nutraceuticals Inc., Shenzhen, China

**Keywords:** GABA, environmental stress, microbial fermentation, neurotransmitter, anabolism, catabolism, biofortification breeding

## Abstract

γ-aminobutyric acid (GABA) is a non-protein amino acid which naturally and widely occurs in animals, plants, and microorganisms. As the chief inhibitory neurotransmitter in the central nervous system of mammals, it has become a popular dietary supplement and has promising application in food industry. The current article reviews the most recent literature regarding the physiological functions, preparation methods, enrichment methods, metabolic pathways, and applications of GABA. This review sheds light on developing GABA-enriched plant varieties and food products, and provides insights for efficient production of GABA through synthetic biology approaches.

## 1 Introduction

**FIGURE 1:**
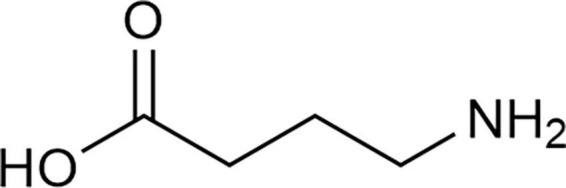
Structural formula of γ-aminobutyric acid (GABA).

γ-aminobutyric acid (GABA), also known as 4-aminobutyric acid (Figure 1), is a non-protein amino acid that naturally and widely occurs in animals, plants, and microorganisms. Its empirical formula is C_4_H_9_O_2_N and the molecular mass is 103.12 g/mol (1). In 1949, GABA was first discovered in potato tubers by Steward et al. (2). GABA is involved in the regulation of growth, development, stress response and other important activities in the life circle of plants. GABA accumulation is mainly affected by environmental factors such as temperature, humidity, and oxygen content (3). In 1950, GABA was found in brains of mammalian mice and rats by Roberts and Frankel (4) and Awapara et al. (5) respectively, where Roberts and Frankel (4) confirmed that GABA in mouse brains was synthesized by α-decarboxylation under the action of glutamic acid decarboxylase (GAD) with glutamic acid (Glu) as substrate. In 1966, GABA was reported by Krnjević and Schwartz (6) as an essential endogenous inhibitory neurotransmitter in the mammalian brain which played a crucial role in maintaining the balance between excitation and inhibition of neural networks. The delicate balance between GABAergic inhibitory neurons and Glu excitable neurons in the brain is the key to the correct functioning of brain. The destruction of this balance is not only related to schizophrenia, autism, epilepsy, and other neuropsychiatric diseases (7) but also related to acquired immune diseases (8, 9). A newly published paper showed that GABAergic regulated the intestinal innate immune response which help to maintain immune homeostasis (10). In addition, GABA was reported to regulate physiological activities such as maintaining blood pressure (11), anti-aging (12), improving liver and kidney functions (13), and ameliorating the executive ability of animals with bidirectional affective disorder (14).

*In vivo* synthesis of GABA is balanced in a healthy adult, however, GABA accumulation turns challenging upon senescence and pressure. Insufficient supply of GABA results in anxiety, fatigue and upset. Therefore, appropriate intake of GABA from diet is of great importance to human health. GABA is widely found in fruits and vegetables, cereals, and edible fungi (Table 1). Fresh strawberry fruit, and fresh lychee fruit, are among the fruits and vegetables, while pumpkin seeds and wheat germ are among the cereals which showed higher GABA content (≧1000 mg/kg). Edible fungi are type of natural healthy food with which are riched in nutrients with delicious taste. A study reported that GABA in the fruiting body of *Flammulina velutipes* (229.7 mg/kg dry base) was relatively riched among the other 20 edible fungi (15). The GABA content in the 11 dried edible fungi range from 15.4 to 4555 mg/kg dry base (16). In addition, the content of GABA in honey and milk was reported low (17). In the mid-1980s, Japanese scientists first developed Gabaron, a GABA-rich tea drink with blood pressure-lowering effect, it was sold as a functional food and attracted widespread attention (18). By 2021, Japan has recorded more than 270 GABA products, and GABA ranks the third place among Japan’s labeled functional food ingredients. In recent years, researchers all over the world are exploring the functional foods enriched with GABA.

**TABLE 1:** Natural γ-aminobutyric acid (GABA) content in foods.

Name		GABA content (mg/kg)	References
Fruits and vegetables	Tomatoes	350−2010	(128, 129)
	Spinach	300−400	(130)
	Broccoli		(131)
	Potatoes	160−610	(132)
	Red mustard leaf	1780	(133)
	Strawberry (fresh)	1560−3640	(134)
	Rambutan (fresh)	719	(135)
	Lychee (fresh)	1700−3500	(136)
	Grape (fresh)	146	(24)
	Longan (dried)	1998	(137)
	Jujube (dried)	150.31−333.37	(138)
Grain	Pumpkin seeds	3710−15530	(139)
	Adzuki	2012	(140)
	Wheat bran flakes	66−99	(141)
	Quinoa flakes		
	Malt powder tablets	258	
	Millet	429	(142)
	Barley bran	948	(143)
	Wheatgerm	1630	(144)
Edible fungi (dried fruiting body)	*Flammulina velutipes*	229.7	(15)
	*Boletus edulis*	202.1	
	*Agaricus bisporus*	125.4	
	*Hypsizygus marmoreus* (brown)	114.1	
	*Clitocybe maxima* (cap)	17.3	
	*Clitocybe maxima* (stipe)	22.7	
	*Pleurotus eryngii*	25.5	
	*Lentinus edodes*	15.4	
	*Grifola frondosa*	17.8	
	*Pholiota nameko*	8.2	
	*Pleurotus citrinopileatus*	17.8	
	*Pleurotus cystidiosus*	37.1	
	*Pleurotus ferulae*	46.7	
	*Pleurotus ostreatus* (Japan)	6.1	
	*Pleurotus ostreatus* (Korea)	23.6	
	*Pleurotus ostreatus* (Taiwan)	Not detected	
	*Pleurotus salmoneostramineus*		
	*Pleurotus eryngii (base)*		
	*Inonotus obliquus*		
	*Auricularia mesenterica*		
	*Boletus with Scallion*	4555	(16)
	*Boletus niger*	3532	
	*Boletus with callion*	2310	
	*Pleurotus geesteranus*	713	
	*Oudemansiella raphanipies*	143	
Other	Honey	4.17−9.77	(145)
	Milk	69−70 mg/L	(146)

There are two highlighted mechanisms in the classical metabolic pathway of GABA: interaction with the GABA shunt (19) and the polyamine degradation pathway (PA pathway) (20), both pathway affected the mitochondrial function. In animals and plants, GABA is a metabolite of glutamate that involved in the conversion of α-ketoglutarate generated by the TCA cycle to produce succinate semialdehyde, which is called GABA shunt. On the other hand, GABA is also generated through PA pathway which involves putrescine and other polyamines (19). GABA in natural food sometimes failed to maintain the physiological needs of human beings. Traditional extraction technology is low efficiency with high cost, which is difficult to supply enough GABA for fortification in food. At present, GABA is mainly enriched through plant metabolism and microbial fermentation (21).

On 27 September 2009, the Ministry of Health of China approved GABA as a new resource food (22). On 31 July 2014, the first industrial standard of GABA was approved and released by the Chinese government. In recent years, GABA enrichment in food has become an industrial and research hotspot. Food-based dietary guidelines (FBDG) by WHO suggested that a healthy diet based on plant foods (cereals, vegetables, and fruits) provide plenty amount of GABA as a supply of natural phytochemical (17). More and more evidences suggest that GABA boosts plant development and exhibits health benefits in human. The physiological functions, metabolic pathways and preparation approaches are reviewed in this paper.

## 2 Effects of GABA on physiological activities in plants

γ-aminobutyric acid exists in plants as a nutrient and signal substance, which participate in regulating metabolism and other physiological activities (23). It can be detected in embryos, cotyledons, roots, stems, leaves, flowers, fruits, seeds, and other organs throughout the development of higher plants at various concentrations (24). GABA is one of the important resistance regulators produced by plants under stress such as crosscutting dynamic plant cell responses when plants are subjected to external environmental stress (25–27).

### 2.1 Effects of GABA on growth and development

As an exogenous additive, GABA improves plant yield and quality through enhancing growth and development. Plant cells produced and secreted GABA spontaneously. Upon the *pop2* defect, GABA signaling is raised in pollen tubes. Different concentrations of GABA (0.99−2.09 μmol/g) promoted or inhibited the growth of *Arabidopsis* pollen tubes. During the research of GABA transaminase (GABA-T) in *Arabidopsis* found that increased levels of GABA caused POP2 activity decreased and *pop2* pollen tubes aberrant growth (28). Similar results were observed in *Picea glauca* pollen tubes: addition of 10−100 mM GABA increased the growth rate of pollen tubes yet addition of over 200 mM GABA retarded the tube growth, indicating that tube elongation was only stimulated by specific concentration of GABA. The calcium-dependent homeostasis of GABA is required for pollen tube growth, where shortage of GABA associated with disorders in actin filament network formation, vesicle trafficking, and cell wall construction (29).

γ-aminobutyric acid restrained iron transportation from roots to shoots in rice seedlings by inducing aerenchyma formation. When treated with GABA or under iron deficiency, the transcription of genes such as *OsIRT1* and *OsNRAMP1* mediated Fe^2+^ transport in the plasma membrane which eventually intensified in roots (30). GABA negatively regulates the formation and growth of adventitious roots of poplar. To prove this assumption, Xie et al. (31) used three methods to increase the accumulation of endogenous GABA in poplar adventitious roots: overexpressing the key gene *PagGAD2* for GABA synthesis; adding exogenous GABA; or inhibiting GABA degradation. The results suggested that root formation was regulated by both exogenous and endogenous factors, including hormones and carbon/nitrogen metabolism, which were associated with the GABA shunt (31). The ripening of plant fruit is highly associated with ethylene regulation (32). The expression of ethylene synthase genes *MaACO1* and *MaACS1* in bananas were up-regulated after GABA treatment, where the ripening period was shorter, indicating that GABA was involved in ethylene regulation (33). Due to the interaction with GABA shunt, tricarboxylic acid cycle (TCA) is affected by GABA concentration together with the activity change of related enzymes. Therefore, GABA serves as an important signal molecule in many pathways which may influence a series of physiological reactions in plants.

### 2.2 Effects of GABA on plant response against stress and adversity

γ-aminobutyric acid participates in direct and indirect defense responses during biotic or abiotic stress (34). A study on *Arabidopsis* found that, insect bite induced rapid local and systemic response of calcium ion cell cycle signal in plants, resulting in the increased of GABA content (35). As a nerve inhibitor, excessive intake of GABA affected the growth and development of insects. It was found that survival (28.57% and 40%) and developmental rates (62.5% and 88.89%) were reduced in larvae of Chordata rosea reared on synthetic diets containing GABA (1.6 and 11.6 μmol GABA/g fresh weight) as compared to the control group. This study suggested that the accumulation of GABA in plants may constitute the first line of defense against herbivores and directly affect the selectivity of insects (36).

The anabolic pathway of GABA in plants is a branch of the TCA cycle, which is sensitive to the oxygen content of the plant growth environment. A hypoxic environment will cause the up-regulated expression of *gad*, a key gene of the GABA synthesis pathway in plants, which promote the accumulation of GABA (37). Under anoxic stress conditions, *Pyrus pyrifolia* showed watercore symptoms where the metabolites succinic acid and GABA were significantly accumulated, accompanied with the up-regulated expression of *PpGAD* gene which indicates the enrichment of GABA (38). Nitrogen deficiency has adverse effects on tree growth. GABA plays a regulatory role at the crossroad of nitrogen metabolism and carbon metabolism. Under limitation of carbon and nitrogen, exogenous GABA affects the on metabolites and transcriptional profiles of Arabidopsis thaliana seedlings (39). Exogenous GABA under also increased the growth of poplar seedlings under low nitrogen conditions (1 mM NH_4_NO_3_) (40). Besides, salt stress inhibits the growth and yield of crops. Exogenous GABA significantly reduced the salt damage index of tomato plants under salt stress, and promoted plant growth, chlorophyll content, and weight gain. The specific mechanism is that GABA reduced the impact of Na^+^ on the growth and yield of tomato plants by preventing Na^+^ from flowing into roots and transporting them to leaves (41). Under excessive heating, the reproductive function of mung bean plants was improved when treated with GABA, indicating that GABA protected plants from heat stress (42). Ramesh et al. (24) interpreted that the mechanism of GABA receptor for regulating plant growth, development and resistance to stress by inhibiting the anion channel of aluminum activated malate transporter family.

## 3 Effects of GABA on physiological and immunity functions in animals

γ-aminobutyric acid functions as an important inhibitor of mammalian neurotransmission by binding to the corresponding receptors. Depending on the sensitivity to agonists and antagonists, GABA receptors are categorized into three types, GABA_A_, GABA_B_, and GABA_C_, where GABA_A_ and GABA_C_ are ionotropic receptors and GABA_B_ is a metabolic receptor (43–45). In mammals, GABA plays a role as neurotransmitter after binding with the receptors, generating multiple effects such as lowering blood pressure, regulating blood glucose, anti-anxiety, and relieving insomnia. Recent studies also reported the function of GABA in tumor immune regulation (46, 47).

### 3.1 Effects of GABA on growth

γ-aminobutyric acid has been shown to be one of the earliest neurotransmitters regulates the nervous system development. Many features of GABA signaling are conserved across species and recapitulated during neurogenesis in the adult brain, indicating that this versatile metabolite is essential for cortical formation (48). Both GAD and GABA_A_ receptors are also expressed in embryonic stem cells and neural crest stem cells in mice, like cortex development, where activation of GABA_A_ downregulated the cell proliferation (49). In *Manduca sexta*, GABA processes olfactory signal in the antennal lobe and shapes development of the olfactory pathway, via both direct and glia- mediated effects (50). The body weight and body mass index of rats with polycystic ovary syndrome were declined when 100 or 500 mg/kg of GABA was added to the diet (51). However, body weight and specific growth rate of Jian carp (*Cyprinus carpio* var. *Jian*) were increased after fed with different concentrations of GABA (30−150 mg/kg), where treatment with 90 mg/kg of GABA resulted the optimal effect (52). GABA affects the feeding behavior of animals thus plays an important role in hypothalamus regulation. Injecting GABA_A_ receptor agonist into different parts of hypothalamus increased the feed intake in animals, which associated with the expression of hypothalamus appetite-related factors, such as neuropeptide Y, cholecystokinin, leptin, and ghrelin (53).

### 3.2 Effects of GABA on blood biochemical indexes

Blood biochemical indexes are common indicators for human health and crucial tools in disease diagnosis (54). In poultry, heterophil-to-lymphocyte ratio is a stress indicator and GABA reported to mitigate the stress response. Study Zhong et al. reported that dietary GABA at 100 mg/kg decreased the corticosterone and heterophil-to-lymphocyte ratio in broiler chickens, but failed to reverse stocking density-induced growth depression (55, 56). Besides, diet supplemented with 100 mg/kg of GABA improved the growth performance of yellow-feathered broilers at 36−49 days of age, as well as downregulated serum intracellular enzyme activities, maintained the organs and intestinal morphology under the high temperature environment. The blood serum biochemical indicators include glucose, total cholesterol, low-density lipoprotein as well as the activities of aspartate-amino-transferase, lactate dehydrogenase and creatine kinase were lower compared to the control group (57).

Several studies shown that GABA-enriched foods contained antihypertensive effect in mammals (Table 2) where fermented products (*Monascus* fermented products) showed better reduction in blood pressure levels than directly obtained from plants. Edible fungi, *Flammulina velutipes* is riched in GABA and showed notable effect in lowering blood pressure. Study showed that a reduction of systolic blood pressure by about 30 mmHg in the hypertensive rats after administration of GABA-enriched *F. velutipes* powder (0.9 mg/kg GABA) (58).

**TABLE 2:** *In vivo* antihypertensive effects of GABA-enriched foods.

Model	Foods	Mode	Antihypertensive effect	References
Spontaneously hypertensive rats	*Flammulina velutipes* powder (0.9 mg GABA/kg)	Single-dose, determine after 8 h	SBP reduction of 30 mm Hg	(58)
	Lactic acid bacteria fermented milk 102 FM (970 mg GABA/L)	Single-dose, determine after 8 h	SBP reduction of 24 mm Hg DBP reduction of 33 mm Hg	(59)
	*Monascus* fermentation products (0.0245 mg GABA/kg)	Single-dose, determine after 8 h	SBP reduction of 30 mm Hg DBP reduction of 20 mm Hg	(60)
	Mulberry leaf water extract (20 mg GABA/kg)	Single-dose, determine after 8 h	SBP reduction of 30 mm Hg	(61)
	Pickled radish (1.36 mg GABA/kg)	single-dose, determine after 2 weeks	SBP reduction of 20 mm Hg	(62)
	Paster fermented by *Aspergillus oryzae idli* (451.7 mg GABA/kg)	Given at every 48 h for 2 weeks, determine after 1 week	SBP reduction of 30 mm Hg	(63)
	Brown rice (1 mg GABA/kg)	Given weekly for 4 weeks, determine after 3 weeks	SBP reduction of 15 mm Hg	(64)

SBP, systolic blood pressure; DBP, diastolic blood pressure.

The mechanism is linked to the GABA postsynaptic neuron receptors, GABA_A_ and GABA_B_, which promotes vasodilation and inhibits sympathetic nerves, thereby reducing blood pressure levels (65–67).

In the cell plasma membrane, GABA activated two types of receptors, among which GABA_A_ receptors open Cl^–^ channels and GABA_B_ receptors are classical G-protein-coupled receptors (GPCRs). Structurally, the GABA_A_ receptors are homo- or hetero- pentamers formed from a selection of 19 known subunits isoforms, yet GABA_B_ receptors are typically formed as a dimer composed of R1 and R2 components (68, 69). Type II diabetes is characterized by insulin resistance and β-cell dysfunction. Thus, β-cell deficit is a common feature in both type I and type II diabetes (70). Maintaining the function of islet cells is one of the key objectives of diabetes treatment. GABA and its substrate Glu mediate a bidirectional pancreatic paracrine signal system located in islet β-cells, and the GABA_A_ receptor is located at islets α-cells excreting glucagon, which associate the GABA signal with islet function (71). Tian et al. (72) found that the combination of proinsulin and GABA alleviated the hyperglycemia symptoms of newly diabetic mice and restored euglycemia. Similarly, the blood glucose level of hypertensive mice treated with GABA-chitosan nanoparticle were significantly lower than control group, the mechanism was linked to the protection of pancreatic islet β-cells and maintaining glucose homeostasis (73). Besides, GABA reduced the blood glucose level of type II diabetes mice model after administration of high-fat diet in combination with streptozotocin, thus reduced the insulin resistance index (74). The GABA mediation over the islet cell network depends on its binding to specific receptors. The GABA_A_ receptor participate in α-cell responses where the alteration in the membrane potentially inhibits the alpha cells’ electric activity by generating more negative potential and blocking the release of glucagon (75).

### 3.3 Effects of GABA on immune response

Activation of GABA receptors inhibits the T cell proliferation which linked to inflammatory diseases (76). Dietary GABA supplementation boosted the mucosal immunity in pathogenic enterotoxigenic *Escherichia coli* infected piglets by releasing the jejunal secretory immunoglobulin A and cytokines such as *interleukin-4*, *interleukin-13*, and *interleukin-17*. The mechanisms might be related to the T-cell-dependent pathway and altered gut microbiota structure and metabolism (77). GABA (100 mg/kg) found to reduce oxidative stress and possess protective effects by recovering ovarian cysts in polycystic ovary syndrome rats (51). When Jian carp (*Cyprinus carpio* var. *Jian*) fish were fed with GABA, their head kidney and spleen expressed higher levels of *interleukin-2*, *interleukin 10*, and *interferon*γ as well as lower levels of *nuclear factor-kappa B*, *interleukin-1*β, and *tumor necrosis factor*α, indicating that GABA involved in activation of immune signaling and prevention against stress (52). GABA supplementation reported to also boost the immune function in livestock, aquatic animals and mammal which could be potentially used as a novel feed additive to prevent stress and disease.

γ-aminobutyric acid is an immune regulatory factor that could improve immunity and to prevent cancer. Xu et al. (78) found that GABA-enriched *Lactobacillus plantarum* GA8 significantly improved the growth performance; promoted probiotic growth in the cecum; inhibited the proliferation of harmful microbe; and improved the serum antioxidant capacity in mice. Besides, GABA enhanced the immune system and alleviated the thermal damage of the 1-day-old Wenchang chicken under the heat stress environment (79). The administration of the mixture of ginseng and GABA alleviated allergic symptoms in the allergen-induced mouse model by reducing histamine and prostaglandin (80). GABA secreted by β-cells activated anti-inflammatory macrophages and inhibited the anti-tumor response of CD8 T cells through the GABA_A_ receptor in colon cancer mice model (81). Lung and colon cancer cells could metabolize and synthesize GABA by abnormal expression of GAD1, a member of the glutamate decarboxylase family, which were shown in many clinical samples of tumor patients, mouse tumor models, and *in vitro* tumor cells. GABA significantly inhibited tumor growth by interfering the expression of GAD1 in tumor cells. The above studies revealed that GABA plays an important role in regulating tumor growth and immune escape which gives a new strategy for cancer immunotherapy (81). The GABA receptors are expressed in immune cells, thereby GABA interact with immune system and display immunoregulatory functions (77).

### 3.4 Anti-anxiety and insomnia relief effects of GABA

Due to the increasing work-related-stress and breakdown of self-tolerance, people nowadays often suffered from mental anxiety which may further developed into insomnia. GABA alleviated anxiety or depression through the neuroendocrine pathway thus improving sleep quality (82). GABA showed positive effects in soothing, compression resistance, and improving sleep quality through EEG detection tests (83). A study found that administration of GABA induced relaxation and reduced anxiety in human under stress conditions (84). Besides, administration of GABA-enriched (26.4 mg/days) defatted rice germ in a clinical study involved 20 female patients ameliorated the autonomic dysfunction and significantly reduced the anxiety symptoms (85). The regulation of GABA on nervous system is linked to mediated the pre-synaptic inhibition of primary afferent fibers in the motor system as well as post-synaptic inhibition of motor neuron.

### 3.5 Other functions

In addition to the above functions, GABA able to ameliorate asthma, mediate weight loss, and relieve alcoholism. A GABA_A_ receptor antagonist-monocrotaline-inhibited the secretion of airway mucus in allergic asthmatic mouse, thereby improved pulmonary function and abated asthma (86). GABA enriched germinated brown rice was reported to promote weight loss, regulate lipid metabolism, relieve oxidative stress, and reduce the risk of cardiovascular disease, where the mechanism was linked to activation of peroxidase receptor γ gene (87). Lateral septum neurotensin neurons (LSNts) played a crucial role in controlling hedonic eating and study showed that GABA signaling mediated the inhibition of LSNts on excessive feeding which could contribute to treatment of obesity (88). GABA-enriched fermented Pueraria also showed anti-alcoholic activity in mice through the drunken latency test (89).

## 4 Enrichment preparation of GABA

γ-aminobutyric acid is a novel food ingredient approved by Ministry in Health of China for its application in food processing (90). Wide application of GABA leads to rapid growth of production to meet the market demand. Traditionally, GABA is extracted from natural plants but the supply is insufficient for fortified food demand. The large-scale production of GABA requires industrial set up. At present, GABA is mainly produced by the following three methods: chemical synthesis, plant enrichment, and microbial fermentation.

### 4.1 Chemical synthesis

**FIGURE 2:**
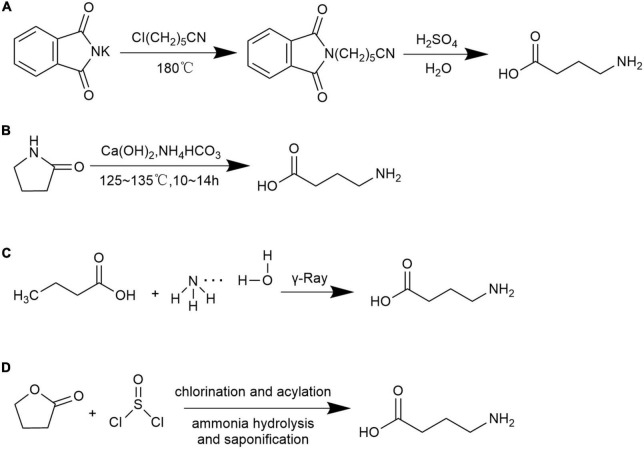
Chemical synthesis methods for GABA. **(A)** γ-Cyanogen chloride method; **(B)** pyrrolidone ring-opening method; **(C)** butyric acid and ammonia water method; and **(D)** γ-butyrolactone and thionyl chloride method.

There are many chemical approaches to synthesize GABA include the following four methods:

A. γ-Cyanogen chloride method (91): under the high-temperature of 180°C, potassium phthalimide (C_8_H_4_KNO_2_) and γ- cyanogen chloroprene (C_4_H_6_CIN) are used as raw materials to react with concentrated sulfuric acid and GABA is obtained through hydrolysis Figure 2A.

B. Pyrrolidone ring-opening method (92, 93): under alkaline conditions, pyrrolidone (C_4_N_7_NO) is used as the starting material, GABA is obtained by hydrolysis and ring-opening reaction under coexistence of calcium hydroxide and ammonium bicarbonate which later processed through decolorization, recrystallization, and purification Figure 2B.
C. Butyric acid and ammonia water method (94): GABA is obtained by using butyric acid (C_4_H_8_O_2_) and ammonia (NH_3_ ⋅ H_2_O) as raw materials under irradiation conditions Figure 2C.
D. γ-Butyrolactone and thionyl chloride method (91): GABA is synthesized by the reaction between butyrolactone (C_4_H_6_O_2_) and thionyl chloride (SOCl_2_), and later processed through chlorination, acylation, ammonolysis, and saponification Figure 2D.

The γ-cyanogen chloride method is a low-cost production but is limited to be popularized in modern manufacture due to the complex processing steps and generation of chemical residues. The pyrrolidone ring-opening method is a non-toxic process and the end products can be easily obtained using cheap raw materials. The polymerization process is mild and safe. However, the pure product is not originated from natural product and failed to be used as food additive. The butyric acid and ammonia water method required few process steps, nevertheless, the process-imposed hazard procedure. The γ-butyrolactone and thionyl chloride method achieves the highest yield, yet with high cost and residual raw materials. The synthesized GABA through the pyrrolidone ring-opening method as well as the γ-butyrolactone and thionyl chloride method were reported as to have purity of 97.1% and 99% with yield of 72.5% and 80%, respectively (91, 92). Yield of GABA produced from γ-cyanogen chloride method and butyric acid and ammonia water methods were barely reported. Although high purity and yield of GABA is obtained though chemical synthesis the limitations of such process involved high cost, safety quality concerns of raw materials, difficulty in controlling reaction conditions and hazard residual chemicals related environmental pollution.

### 4.2 Plant enrichment

γ-aminobutyric acid is synthesized primarily through the L-glutamate (L-Glu) by GAD catalysis in higher plants. This pathway is related to various internal and external stimuli to plant tissues, including high- or low-temperature stress, salt stress, hypoxia stress, and mechanical damage (95). The plant enrichment method is to increase GABA content in plants through environmental stress.

Studies showed that GABA content in grains (such as wheat, barley, and rice) or legumes (such as soybean) can be enhanced under the salt, soaking, and hypoxia stress treatment. Recent years, non-thermal processing technologies provide new safe and effective stress approaches to enrich GABA in plants, such as high static pressure, ultrasonic, and electrolytic water. Through the GABA content of stress- treated plants are in the ranged of reached 143−2850 mg/kg, where the rice germinated at 30°C for 20 days recorded the most significant enrichment effect with GABA 2850 mg/kg content (Table 3). At present, several GABA-enriched plant foods are available in the market include bread, bean sprouts, and rice vinegar.

**TABLE 3:** Different GABA enrichment approaches in plants.

Product	Enrichment method	Treatment conditions	GABA concentration (mg/kg)	References
Glutinous Barley without Hull	Soaking and anaerobic treatment	Soak in 50 mmol/L sodium acetate buffer (pH 6.0) for 8 h, germinate at 15°C for 48 h, and then anaerobic treat for 12 h	143	(147)
Wheat	Hydration anaerobic treatment	After five times of hydration treatment, the wheat moisture reaches 35%, and nitrogen is added to maintain the pressure of 20 Pa for anaerobic treatment for 24 h	456.5	(148)
	Hydration heat treatment	Wheat moisture reaches 35%, blowing at 120−140°C for the 30 s, wheat grain temperature reaches 50−60°C, and then cooling to 27°C	474	
Barley	Soaking treatment	Soak at 15°C, imbibe for 48 h, and germinate for 72 h	529	(149)
Wheat	Sonication treatment	After germination, 25 kHz, 28°C, 72 h ultrasonic treatment for 30 min	497.2	(150)
Soybean	Salt stress treatment	Soak in 100 mmol/L NaCl solution for 12 h at 25°C	2699.3	(151)
Bean sprout	Salt stress treatment	33.3°C, 133.5 mmol/L NaCl solution culture time 5.5 days	1205.24	(152)
Soybean sprouts	Sonication treatment	25°C, 300 W treatment for 30 min	1192.9	(153)
Soybean	Soaking treatment	30°C, soaking for 4 h, hypoxia stress for 2 days	2240	(154)
Millet	Salt stress treatment	25°C, 100 mmol/L NaCl solution salt stress treatment for 48 h	319.2	(155)
Rice	Salt stress treatment	Soak in 30 mmol/L NaCl solution at 20°C for 48 h	1138.2	(156)
Brown rice	Salt stress treatment	33.3°C, soak in 133.5 mmol/L NaCl solution for 72 h	1217.14	(157)
Rice bran	Hypoxic stress treatment	40°C, anaerobic culture under nitrogen for 8 h	1715	(158)
Rice	Treatment with different germination times	Germination at 30°C for 20 days	2850	(159)
Germinated soybean	Lactic acid bacteria fermentation treatment	MRS culture solution, at 30°C, 150 rpm, fermented soybean with lactic acid bacteria for 24 h	424.67	(160)

### 4.3 Microbial fermentation

Microbial fermentation of GABA typically use Glu, MSG, or substances rich in Glu as raw materials, and *Saccharomyces*, *Lactobacillus*, *E. coli*, and *Aspergillus* are the microbial strains commonly used for the fermentation (96). Early researches of microbial fermentation focus mainly using *E. coli* for the production of concentrated GABA. For example, Plokhov et al. (97) used *E. coli* with high GAD activity to produce GABA, and Zhao (98) immobilized *E. coli* in the cell immobilization system where embedded calcium alginate allows the transformation of intracellular Glu into GABA.

In recent years, safer microorganisms such as *Lactobacillus*, *Aspergillus*, and *Saccharomyces* are widely used in the production of GABA food. The content of GABA obtained by microbial fermentation is higher than plant enrichment with the range of 0.191−22.373 g/L, where *Monascus* fermentation recorded the highest yield (Table 4). At present, GABA produced through microbial fermentation has been further processed into foods such as bread, yogurt, lactobacillus beverage, and jelly in the market.

**TABLE 4:** Enrichment of GABA by microbial fermentation.

Strain	Enrichment method	Treatment conditions	GABA concentration (g/L)	References
*Lactobacillus bulgaricus* L2	UV mutagenesis	Irradiation by 18 W ultraviolet lamp, at 45 cm for 50 s	1.3392	(161)
*Streptococcus thermophilus*	Fermentation	Culture in MRS medium at 37°C for 48 h	2.8	(162)
*Lactobacillus plantarum* CICC 6238	UV mutagenesis	Irradiation by 30 W ultraviolet lamp at 25 cm for 9 s	1.196	(110)
*Lactobacillus plantarum* FNCC 260	Fermentation	Culture in MRS medium at 37°C for 60 h	0.809	(163)
*Lentinula edodes*	Malolactic fermentation	In MRS medium, shiitake stalk is activated once at 37°C and 150 rpm/min for 16−20 h, and co-activated for 3 times	0.191	(164)
*Lactobacillus Plantarum Lac.1*	Co-culture	*Monascus*SM048 at 30°C, 200 rpm/min, after 7 days of culture,	0.52	(165)
*Monascus* SM048		*Lactobacillus* is inoculated and incubated at 30°C for 120 h		
*Monascus*	Fermentation	Shake-flask at 29°C, 200 rpm/min for 6 days	22.373	(166)
*Monascus* M-6	Fermentation	fermentation at pH 5.0, 40°C, 120 rpm/min, for 48 h	7.826	(167)
*Saccharomyces* mutant	Fermentation	Inoculate at concentration of 4% and incubate at 30°C, 220 rpm for 4 days	2.588	(168)
*Candida glabra* GPT-5-11	Fermentation	Incubate at 37°C, pH 6.5 for 48 h	2.58	(169)
*Monascus* CH-1	Fermentation	Inoculate at concentration of 17% and incubate at 30°C for 9 days; the substrate is added with 0.10 g of sodium glutamate	1930 (mg/kg)	(170)
*Lactobacillus brevis* GABA100	Co-culture		2.5 × 10^8^ (CFU/mL)	(171)
*Bifidobacterium* BGN4		Incubate at 30°C, 195−200 rpm, anaerobic condition for 6 days	8.7 × 10^6^ (CFU/mL)	

## 5 Anabolism and catabolism of GABA

Similar to the secondary metabolites, the *in vivo* metabolism of GABA consists of anabolism and catabolism. There are two main metabolic pathways of GABA: GABA branched pathway and the polyamine degradation pathway. Among them, the GABA-branched pathway has been widely reported. Since the GABA metabolic pathways are closely related to the antioxidant system, metabolic shunting of GABA is considered as a necessary defense strategy when respiration and the TCA cycle are inhibited or impaired under stressful conditions.

### 5.1 Metabolic pathways of GABA

The metabolic pathway of GABA was first reported in rats by Bessman et al. (99). Because it is coupled with the TCA cycle, it is called GABA shunt and is accompanied by electron transmission of the respiratory chain. In the TCA cycle, α-Ketoglutarate is catalyzed by glutamate dehydrogenase (GDH) to produce Glu, and then under the action of GAD, an irreversible decarboxylation reaction takes place on the α site of Glu to generate GABA. This step is accompanied by the consumption of a proton and the release of one molecule of CO_2_. GABA generates succinic semialdehyde (SSA) under the action of GABA-T. SSA generates succinate (Suc) through the catalysis of succinic semialdehyde dehydrogenase (SSADH) and then enters the TCA cycle again. At the same time, NADP receives H^+^ and converts it into NAD^+^ (19). The GABA branch pathway is reported in microorganisms (100), mammalian brains [4,5], and most higher plants (101).

In *Arabidopsis thaliana*, there is another GABA metabolic pathway- the PA pathway. Polyamines are a category of small molecule ammonia-containing bases in plants, mainly including putrescine (Put), spermidine (Spd), and spermine (Spin). They exist in roots, stems, leaves, flowers, fruits, and other organs, participate in the whole process of plant growth and development, and are closely related to stress resistance. In the PA pathway, Glu is converted into α-Ketoglutarate by α-Ketoglutarate dependent GABA transaminase (GABA-TK) and ultimately provides ORN. ORN is transferred to the cytosol and subsequently converts to GABA via PA pathway (27). ORN is transferred to the cytoplasm and forms putrescine (Put) and other amines under the action of ornithine decarboxylase (ORD). Diamines or polyamines are respectively catalyzed by diamine oxidase (DAO) or polyamine oxidase (PAO) to produce 4-aminobutyraldehyde. Finally, GABA is generated through a dehydrogenation reaction catalyzed by 4-aminobutyraldehyde dehydrogenase (AMADH). PA pathway intersects with the GABA branch pathway, and then the metabolites enter the TCA cycle (102).

**FIGURE 3:**
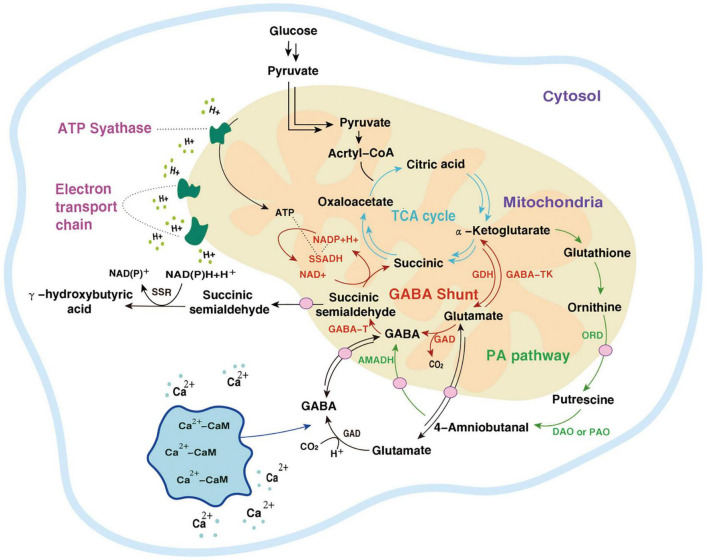
Two metabolic pathways of GABA. 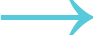, the TCA cycle; 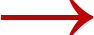, the GABA shunt; 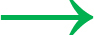, the PA pathway; 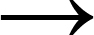, other pathways; 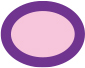, a transporter; 
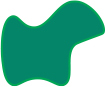
, the electron transport chain.

**FIGURE 4:**
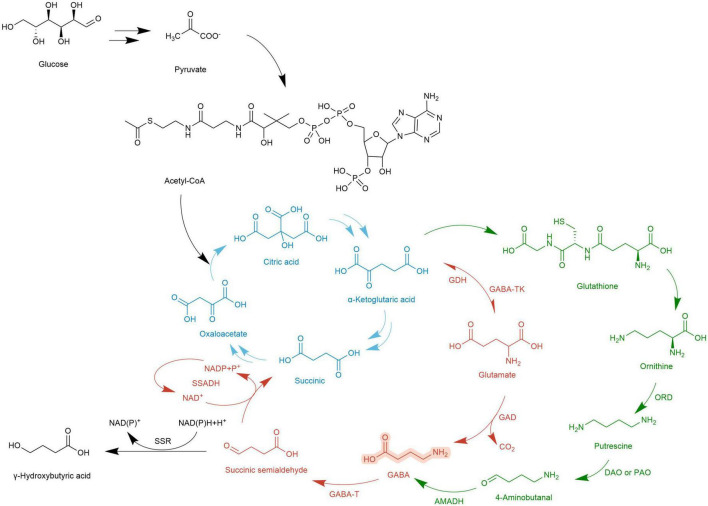
Metabolic map of GABA synthesis and GABA degradation. 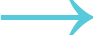, TCA cycle; 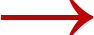, GABA shunt; 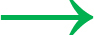, PA pathway; 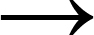, Otherway.

The GABA pathway and PA pathway mainly occur in mitochondria. In the PA pathway, the conversion of Put to 4-aminobutyraldehyde takes place in the cytoplasm. Suc and NADP are electron donors in the mitochondrial electron transport chain whose terminal product is ATP (103). The intermediate SSA of the TCA cycle can also be transported to the cytoplasm by subcellular organelles and is catalyzed by succinic semialdehyde reductase (SSR) γ to produce hydroxybutyric acid (GHB). In addition, calcium ions (Ca^2+^) located in the cytoplasm combine with calmodulin (CaM) to improve the activity of GAD. After Glu is transformed into GABA in the cytoplasm, it is transported to mitochondria and then enters the GABA branch pathway (104). In macro-fungi, the biosynthesis and storage of complex natural products are characterized by spanning multiple types of subcellular compartments, and even across different tissues and organs. Each subcellular compartment provides a unique physiological environment. Referring to the location where the metabolic processes take place, the metabolism of GABA (even as a small molecule with simple structure) also indicates the characteristics of compartmentalization (Figures 3, 4).

### 5.2 Key enzymes in the GABA metabolic pathway

The GABA pathway is the most important pathway of GABA metabolism. Three key enzymes play an important role in this pathway: GAD, GABA-T, and SSADH (19). Among them, GAD catalyzing the decarboxylation of Glu to produce GABA is the most studied.

#### 5.2.1 GAD

Glutamic acid decarboxylase belongs to the 5′−phosphate pyridoxal (PLP) dependent enzyme family, is the key enzyme of GABA synthesis which catalyze the decarboxylation of L-Glu to GABA. The process of GAD catalyze to GABA can be divided into three stages (105): (1) In the absence of substrates, ε-NH_2_ is covalently bonded with Schiff base to form an internal aldehyde imine structure; (2) When L-Glu enters the catalytic active pocket, PLP and α-NH_2_ combines to form an external aldehyde imine, thereby activating the decarboxylation reaction to form a quinone intermediate; (3) The quinone intermediate is structurally unstable, which immediately combines with PLP to form aldehyde imine structure and releases GABA.

Glutamic acid decarboxylase exists widely in animals, plants, and microorganisms. The structure and properties of GAD across species are different. The optimum pH for the catalytic reaction of GAD derived from microorganisms is usually 3.8−5.0. The enzyme activity is strong in an acidic medium, and it decreases or even disappears when the pH is neutral or alkaline; The optimum reaction temperature of GAD is related to the growth temperature of microorganisms, mostly at 30−50°C (106). Shirasaka et al. (107) studied the enzymatic properties of GAD in the fruiting body of *F. velutipes*. After separation and purification, the molecular weight of GAD obtained was 30 kDa under optimal pH value of 6, and optimal temperature at 28°C. The activity of GAD was stable below 50°C. Furthermore, Hua et al. (108) improved the thermal stability of *Lactobacillus brevis* GAD by consensus mutation technology to enhance the enzymatic properties.

With the development of molecular biology techniques, the *gad* gene in many plants and microorganisms, such as *Arabidopsis thaliana*, tomato, *E. coli*, and *Lactobacillus*, has been cloned and expressed, and the biochemical characteristics of the corresponding GAD protein have also been preliminarily clarified. Two *gad* genes were cloned from *E. coli*, *gadA*, and *gadB*, with the nucleotide similarity of 98%. Only five different amino acid residues were found between the two GAD proteins (109). Turano et al. (110) cloned and expressed the *gad* gene of *Arabidopsis thaliana*, and found that the two gad-encoded proteins were CaM-binding proteins, and their activities were regulated by Ca^2+^/CaM (111). The *Lactobacillus* K285 with high GABA production capacity was isolated and identified from Korean pickles, which could be overexpressed and purified in *E. coli* (112). A DNA fragment containing a complete *gadA* site with high GABA production capacity in *Lactobacillus brevis* NCL912 was successfully obtained by single primer PCR (113). In recent years, a famous study on GABA biosynthesis was conducted in tomatoes. A key factor of the GABA metabolic pathway in tomatoes−*SlGAD3*−was identified by RNAi technology. Overexpression of *SlGAD3* increased the mRNA levels in 20−200 fold in mature green and red tomato fruits which in turns significant increased the GABA content, with no abnormality found during the development of fruits and vegetative organs (114). On this basis, the research team overexpressed the fruit-specific promoter with 87 nucleotides missing at the C-terminal and the coding sequence of *SlGAD3*. The results showed that, as compared with the mutant strain with single *SlGAD3* overexpression, the new gene editing further improved the GABA content of red mature tomato fruits (115).

#### 5.2.2 GABA-T

γ-aminobutyric acid-transaminase belongs to the class III pyridoxal phosphate-dependent aminotransferase family, which catalyzes the reaction of pyruvate and GABA to generate SSA and alanine and plays a key role in GABA catabolism (116). The GABA-T gene *GabT* was cloned from the *Bacillus thuringiensis* G03 strain, and its catalytic activity was confirmed by expression in *E. coli* (117). Under hypoxia conditions, the activities of GAD, DAO, and PAO in fresh tea increased significantly, while the activities of GABA-T decreased significantly, leading to the accumulation of GABA (118). Additionally, downregulated expression of GABA-T in rice by RNAi promoted the accumulation of GABA in rice (119). After the ultrasonic treatment, the expression of the *gad* gene in fresh coffee leaves was activated by, inhibiting the GABA-T activity, and increasing GABA content (120, 121). GABA-T enzyme has two isomers which are pyruvate-dependent GABA-T (GABA-TP) and α-ketoglutarate-dependent GABA-T (GABA-TK). Although there was a strong similarity between the two isomers, only GABA-TP was identified in tomatoes (122). Li et al. (123) selected *GABA-TP1*, *GABA-TP2*, *GABA-TP3*, *CAT*, and *SSADH* genes involved in GABA metabolism as target genes for editing, and found that most of the mutant plants were sterile, only GABA-2 (the mutant plant of *GABA-TP1*) and GABA-3 (the mutant plant of *GABA-TP3*) could bear fruits. The contents of GABA-2 and GABA-3 in green fruits recorded at 1028 mg/kg and 1097.2 mg/kg respectively, which were 1.34 and 1.43 times higher than those of wild type plants respectively. The contents of GABA-2 and GABA-3 in red fruits recorded at 758.3 mg/kg and 898.8 mg/kg, respectively which were 2.95 and 3.50 times higher than those of wild type plants. The activities of GABA-TP and GAD enzymes in fruits were significantly lower than those in wild type plants, indicating that the accumulation of GABA showed negative feedback to its anabolic pathway (123).

#### 5.2.3 SSADH

Besides GABA-T, GABA catabolic enzymes also catalyze the oxidation of SSA to produce Suc. Jia et al. (124) studied the enzymatic properties of SSADH and the enzyme activity was greatly affected by Cu^2+^ under the optimal reaction temperature of 37°C with optimal pH of 7.5. Frank et al. (125) found that the peroxide content in the *Arabidopsis thaliana SSADH* mutant was increased and the growth development was affected, which could be associated the accumulation of SSA and GHB. According to a study focusing on GABA-T-SSADH coupling activity of the GabT-GabD protein, NCgl2515 participated in the catabolism and utilization of GABA with GABA-T activity except for GabT in *Corynebacterium glutamicum* (126).

## 6 Prospects

γ-aminobutyric acid attracted research and industrial interest due to its versatility over the last few decades with the multiple health benefits and promising applications in food industry. Besides, targeted nutrient enrichment is also becoming a popular goal of crop breeding (127). China launched the Harvest Plus China project in 2004 in order to meet the breeding demands of high-yield and high-quality crop varieties and a few new crop varieties have been successfully developed, such as zinc-fortified wheat and rice; iron-fortified wheat; rice and corn; vitamin A fortified wheat; corn and sweet potato; GABA-fortified rice; and sulforaphane-fortified Chinese cabbage. GABA-enriched plant and microorganism variety might become a new future direction for breeding.

γ-aminobutyric acid-enriched foods can be produced through biosynthetic microbial approach which is more effective, cost saving and eco-friendly. More research on the benefits of GABA in humans and microbes can be further explored in the future. GABA-rich fermented food offers a promising approach to better control of hypertension. Edible fungi are enriched in essential amino acids and a variety of bioactive compounds with high protein and low calories. To achieve the “nutrient enhancement” breeding goal in edible fungi, high-yield mechanism of GABA based on metabolic regulation network is recommended in order to provide effective strategy to optimize germplasm, efficiency of breeding program and facilitate nutrient-enhanced product development. The effects of GABA in edible fungi can be further explored by knocking out the *GAD* genes or blocking the GABA biosynthesis in the future.

## Author contributions

ZH wrote the original draft. CH drafted and revised the manuscript. BD supervised the literature review and manuscript writing. TY revised and improved the manuscript. ZY and ZX revised the manuscript. WY conceived the project, organized the writing, and revised the manuscript. All authors contributed to the article and approved the submitted version.
